# Attrition and generalizability in longitudinal studies: findings from a 15-year population-based study and a Monte Carlo simulation study

**DOI:** 10.1186/1471-2458-12-918

**Published:** 2012-10-29

**Authors:** Kristin Gustavson, Tilmann von Soest, Evalill Karevold, Espen Røysamb

**Affiliations:** 1 Norwegian Institute of Public Health, Division of Mental Health, Department of Child and Adolescent Mental Health, P.O. Box 4404, Nydalen, NO-0403, Oslo, Norway; 2 Department of Psychology, University of Oslo, P.O. Box 1072, Blindern, NO-0316, Oslo, Norway

**Keywords:** Longitudinal studies, Public health, Attrition, Bias, Simulation

## Abstract

**Background:**

Attrition is one of the major methodological problems in longitudinal studies. It can deteriorate generalizability of findings if participants who stay in a study differ from those who drop out. The aim of this study was to examine the degree to which attrition leads to biased estimates of means of variables and associations between them.

**Methods:**

Mothers of 18-month-old children were enrolled in a population-based study in 1993 (N=913) that aimed to examine development in children and their families in the general population. Fifteen years later, 56% of the sample had dropped out. The present study examined predictors of attrition as well as baseline associations between variables among those who stayed and those who dropped out of that study. A Monte Carlo simulation study was also performed.

**Results:**

Those who had dropped out of the study over 15 years had lower educational level at baseline than those who stayed, but they did not differ regarding baseline psychological and relationship variables. Baseline correlations were the same among those who stayed and those who later dropped out. The simulation study showed that estimates of means became biased even at low attrition rates and only weak dependency between attrition and follow-up variables. Estimates of associations between variables became biased only when attrition was dependent on both baseline and follow-up variables. Attrition rate did not affect estimates of associations between variables.

**Conclusions:**

Long-term longitudinal studies are valuable for studying associations between risk/protective factors and health outcomes even considering substantial attrition rates.

## Background

Longitudinal studies are important in public health research for identifying risk factors related to negative health outcomes. However, a major concern in such studies is that the longer the follow-up period, the higher the chances are for drop-out [1]. Attrition rates from 30 to 70% are often reported [2-7]. Thus, it is important to study the effect of attrition on the generalizability of findings from long-term longitudinal studies. One of the aims of the current paper is to examine attrition from a 15-year population-based longitudinal study (TOPP study) initiated in 1993 to investigate development in children and their families. The TOPP study includes information about socio-demographics, psychological factors in the children and their mothers, and social relationships.

Differences in mean levels of variables between those who drop out and those who stay in a study do not necessarily imply that there are differences in associations between variables [7,8]. Even though a major concern in public health research is associations between risk/protective factors and later health outcomes, most attrition studies only examine the degree to which those who stay and those who drop out of a study are comparable in terms of mean levels. The current study also examines possible effects of attrition on estimates of associations between variables.

Even if those who stay and those who drop out of a study are similar at baseline, they may be different at the time of follow-up. Researchers seldom have information about how people who drop out would have responded if they had stayed in the study. Therefore, real life studies are generally not suited to examine effects of attrition that is dependent on follow-up variables. Thus, in the current study we use a computer simulation to examine effects on parameter estimates of attrition that is dependent on both baseline and follow-up variables.

### Baseline predictors of attrition

Previous research has shown that different factors can influence drop-out rate. Socio-demographic variables, such as low educational level, being out of work, and not being married, are typically related to increased risk of non-response and attrition in epidemiological studies [2,4,5,8-12]. In addition, unhealthy life style factors, such as smoking, high alcohol consumption, and physical inactivity, are related to non-participation and attrition [8,11-13].

High levels of psychological distress can predict attrition in high-risk populations, such as psychiatric outpatients and former hospitalized patients [3,14]. In population-based studies, psychological distress has been found to have no effect or a weak to moderate effect on attrition after adjusting for other variables [2,4,9,10]. Attrition may also be related to social factors, such as support from spouse or friends, and child’s characteristics. Poor relationship quality is an important predictor of mental health problems [15]. However, social networks and support did not predict attrition in a 15-year follow-up study [5], and marital satisfaction and spousal support did not predict attrition in a job satisfaction study [6]. More knowledge is needed about the association between attrition and psychological as well as social factors.

Studies with high-risk populations found that externalizing problems and psychopathology in general among children were associated with a higher risk of parents dropping out [16,17], whereas child characteristics such as temperament, anxiety, and attention problems did not predict attrition in population-based studies [18,19]. It may be that the ways different factors affect attrition are dependent on whether the original sample was drawn from a high-risk population. In general, we need more knowledge about psychological variables and family characteristics, since previous research on these topics is divergent and relatively sparse.

Continued participation in a study 10–15 years later may depend on factors other than those related to participation in a shorter time perspective. For example, some mental disorders were differentially associated with attrition in a one-year compared to a 15-year follow-up of a geographical sub-sample in the same study [5,9]. Both short-term and long-term attrition should therefore be studied to examine whether samples in long-term studies become increasingly biased over time.

### Baseline associations between variables

Public mental health research typically examines how variables such as demographics, social relationships, life stress, and personality predict mental health. Further studies are needed to examine possible baseline differences in associations between variables related to participants who stay compared to those who drop out of longitudinal studies.

### Effects of attrition dependent on follow-up variables

Those who stay can be more different from those who drop out of a study at the time of follow-up than at baseline, suggesting that attrition is dependent on follow-up variables. This further implies that attrition is dependent on variables with missing data because the researcher generally only has information on follow-up variables from those who stayed in the study. Therefore, it is not possible to control for sample biases that are related to attrition dependent on follow-up variables. Statistical techniques to account for missing data, such as full information maximum likelihood and most current forms of multiple imputation, are less efficient when missingness is dependent on variables with missing data than when it is only dependent on variables with information from all participants [20]. Therefore, the effect of attrition dependent on follow-up variables needs to be studied.

Computer simulation studies can be used to examine the effect of attrition that is dependent on unobserved variables. Researchers in simulation studies know the true parameter values in the population [21-23]. Scenarios where attrition is dependent on unobserved variables can be simulated by generating data sets where attrition is dependent on variables with missing values. Parameter estimates obtained from such samples can then be compared to the known true population parameters. Previous simulation studies have examined the effects of non-random attrition on estimates of effects of interventions, estimates of odds ratios, and of cumulative probabilities [21,24,25]. Such studies have typically compared the effect of attrition that is completely random to non-random attrition [21,24]. We extend current knowledge by examining the effect of attrition under conditions with different levels of dependency between risk of attrition and predictors as well as follow-up variables.

### Aims

The general aim of the current study was to examine the effects of attrition in long-term longitudinal studies. Specific aims were to examine baseline predictors of short-term (one-year follow-up) and long-term (15-year follow-up) attrition and to examine potential differences in baseline correlations between those who stayed and those who dropped out of a 15-year study. The last aim was to perform a computer simulation study to examine the effect of attrition that is dependent on unobserved variables.

## Method

### The real life study

#### Design and sample

We used data from the Tracking Opportunities and Problems (TOPP) study, which is a longitudinal study that started in 1993. At 19 different community health care centers in eastern Norway, mothers attending with their 18-month-old child were asked to fill out a questionnaire (baseline). Over 95% of Norwegian families attended such health care centers. Of the parents invited to participate, 87% (N= 929) completed the questionnaire; 913 were mothers, and 16 were fathers. The women received a new questionnaire when their child was 2.5 years old (one-year follow-up) and 4 years old, and they were asked to return it to the staff when attending the center for a routine check of their child. New questionnaires were sent by mail to the women at four additional time points, when the children were 8–9, 12–13, 14–15 and 16–17 years old (15-year follow-up). More information about the design and sample can be found elsewhere [26,27]. Women’s data from baseline, one-year follow-up, and 15-year follow-up were used in the current study. Examining attrition from baseline to each of the six follow-up waves represents a large number of analyses and is beyond the scope of the current study. Attrition at one- and 15-year follow-ups was chosen to represent short-term and long-term attrition, respectively.

The research project was approved by the Norwegian Data Inspectorate and the Regional Committee for Medical Research Ethics.

#### Measures

##### Socio-demographic variables

Socio-demographic variables at baseline included age, educational level, marital status, employment status, and perception of personal financial situation. Women reported their educational level (number of years in school) at baseline on a scale ranging from 1 to 8 where 1 represented 7 years or less in school and 8 represented 4 or more years in university/college. Employment status was a dichotomous variable with 1 representing currently working/studying or on maternity leave, while 0 represented not working because of unemployment or health problems. Financial situation was rated on a 5-point scale with 1 being “We are managing very poorly financially” and 5 being “We are managing very well financially” [28].

##### Psychological variables

Women’s psychological distress was assessed at baseline using the Hopkins Symptom Checklist-25 (HSCL-25) [29,30]. One item (about sexual interest) was left out because of its sensitive nature. The reliability and validity of the HSCL are well established [31,32]. Cronbach’s alpha was 0.90.

Women’s temperament was assessed at baseline with the Emotionality, Activity, and Sociability Temperament Questionnaire (EAS) [33]. Emotionality (fear, anger, and distress) is measured with twelve items, and sociability and activity with four items each. All three temperament traits are measured on a 5-point scale ranging from ‘not typical’ to ‘very typical.’ An examination of the factor structure, reliability, and stability of the EAS with the TOPP data set has shown that the temperament scales have acceptable psychometric properties [34]. Cronbach’s alphas at baseline were 0.73, 0.65, and 0.54 for emotionality, activity, and sociability, respectively.

##### Child temperament

The child version of the EAS [33] was used to measure children’s temperament (emotionality, activity, sociability, and shyness) at baseline. The EAS has been shown to be a sound measure of young children’s temperament [35,36]. Cronbach’s alphas were 0.62, 0.69, 0.48, and 0.72 for emotionality, activity, sociability, and shyness, respectively.

##### Chronic stressors

The women answered 11 questions about long-lasting stressors related to child care, raising children, finances, household, employment, their own and their spouses’ health, and children’s health problems [26,37]. The items were rated on a 4-point scale, and the mean score of the 11 items was used as an index of chronic stressors. Cronbach’s alpha for this scale was 0.68.

##### Social variables

The relationship quality index at baseline included five questions about emotional support from spouse/partner (feeling attached to partner, whether partner valued one’s opinions, helping and supporting each other, feeling of belonging, and feeling left out even at home) [26,38]. Mean scores on these five items were computed. Cronbach’s alpha was 0.76. The women also completed a social support scale that consisted of six items on emotional support from family and friends [26,38]. Cronbach’s alpha was 0.72.

#### Statistical analyses

Logistic regression analyses were performed to assess how variables from the baseline questionnaire predicted drop-outs at one- and 15-year follow-ups. Predictor variables were standardized (setting standard deviation to 1 and mean to 0) such that odds ratios could be compared as measures of effect size. Age, employment status, and marital/cohabitation status were not standardized because of the interval scale or dichotomous nature of these measures. The distributions of the variables Chronic stressors and HSCL were significantly different from normal (skewness value > two times standard error of skewness) [39]. These variables were thus log-transformed for use in correlation and linear regression analyses.

To examine whether those who later dropped out differed from those who stayed in regard to associations between variables at baseline, a series of regression analyses with interaction terms was performed. Thus, associations between 15 potential predictors and women’s psychological distress (HSCL) at baseline were compared for those who remained in the study and those who later dropped out.

Power analysis was conducted with the G*Power 3.1.2 program [40,41]. For the simple logistic regression analysis, an odds ratio of 1.28 in the population could be detected with a probability of .80 for short-term attrition, while an odds ratio of 1.21 could be detected with a probability of .80 for long-term attrition.

Since multiple tests were performed, the significance level was Bonferroni corrected for the number of tests performed so that the alpha level for each individual test was set at 0.003. In addition, basic uncorrected 95% confidence interval and uncorrected significance level for the odds ratios are reported.

### The simulation study

A Monte Carlo simulation study was performed in Mplus [22]. Such studies are often used to investigate how statistical estimators work under different conditions [22,23] and can be used to examine how parameter estimates are affected by non-random attrition [21]. Data were randomly drawn from a theoretical population with certain researcher-defined parameters [42]. The true parameter values were thus known and could be compared to parameter estimates obtained from generated samples with non-random attrition.

The external Monte Carlo approach was used, meaning that the data sets were generated in a first step and then analyzed in a second step [22].

**Figure 1:**
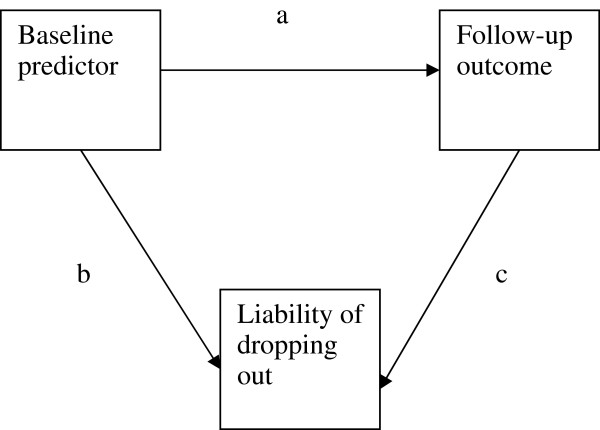
**Theoretical model guiding the simulation study.** Note: The association between *Baseline predictor* and *Liability of dropping out* is the sum of the direct path (**b**) and the indirect path via *follow-up outcome* (**a** x **c**). The association between *Follow-up outcome* and *Liability of dropping out* is the sum of the direct path (**c)** and the indirect path via *Baseline predictor* (**a** x **b**). This is accounted for in the analyses.

Two study variables, *Baseline predictor and Follow-up outcome*, were created (see Figure 1). This was done to mimic a risk factor measured at one time point and a health outcome measured at a later time point in a real life longitudinal study. Population means were set to zero and population variance to one for both variables. These variables were modeled to be normally distributed in the population. The population association between the two variables (Path a in Figure 1) was first set to β =.10, which is a small effect size according to Cohen [43]. In a second part of the simulation study, the association between *Baseline predictor* and *Follow-up outcome* was set to β =.30, which is a medium effect size. This was done to examine the effect of attrition under different levels of population associations between a baseline predictor and a later outcome.

Response or non-response in a study can be seen as a categorical manifestation of an underlying dimensional liability of not responding. In the current study, the risk of attrition was modeled as a continuous normally distributed tendency (*Liability of dropping out*). Different sets of samples were generated where the dependency between *Liability of dropping out* and *Follow-up outcome* (Path c in Figure 1) varied from zero to a medium-to-large effect size (from β =.00 to β =.40). In addition, dependency between *Liability of dropping out* and *Baseline predictor* (Path b in Figure 1) also varied from zero to a medium-to-large effect size (β =.00 to β =.40). We thus simulated situations with different combinations of dependency between attrition and both *Baseline predictor* and *Follow-up outcome*, including a situation with completely random attrition. In the real life TOPP study, drop-out was measured as a dichotomized variable, and hence ORs were reported. The βs used in the simulation study correspond roughly to the following ORs (depending on attrition rate): β =.00 equals OR =1; β =.10 ≈ OR = 1.22; β =.20 ≈ OR =1.49; β =.30 ≈ OR =1.82; β =.40 ≈ OR = 2.23.

For each condition, 500 data sets were generated [21,44]. Each data set consisted of 1000 observations to mimic the baseline sample size in the TOPP study. Analyses were then run on sub-samples with different attrition rates. First, all observations with scores up to .52 standard deviations above the mean on *Liability of dropping out* were included in analyses to model a 30% attrition rate. Second, only observations with scores below the mean on *Liability of dropping out* were included. This modeled the attrition rate of 50% in the real life TOPP study, as well as several other studies. Third, only observations with scores up to .52 standard deviations below the mean on *Liability of dropping out* were included, thus modeling a 70% attrition rate. This procedure mimicked situations where only those who remain in a study are included in analyses (complete case analysis/listwise deletion). Results from these analyses were then compared to the known true population means and associations.

## Results

### The real life study

At one year follow-up, 155 women (17% of the sample) had dropped out, whereas 56% of the sample (514 of 913) had dropped out between baseline and the 15-year follow-up. Attrition was mainly because of refusal to participate, while death or failure to locate was very rare. Table 1 reports baseline descriptive statistics for all continuous variables.

**Table 1:** Mean values and standard deviations for baseline variables

**Variable**	**Mean**	**Standard deviation**
*Women:*		
Age	29.9	4.77
Family finances (1–5)	3.58	0.80
Education (1–8)	5.94	1.45
HSCL (1–4)	1.35	0.34
Emotionality (1–5)	2.53	0.51
Sociability (1–5)	3.74	0.59
Activity (1–5)	3.01	0.70
Emotional support (1–5)	4.45	0.69
Emotional support from friends and family (1–5)	4.18	0.68
Chronic stressors (1–4)	1.30	0.31
*Children:*		
Activity (1–5)	4.32	0.55
Sociability (1–5)	3.95	0.52
Emotionality (1–5)	2.79	0.61
Shyness (1–5)	2.02	0.63

### Baseline predictors of attrition

Table 2 shows that drop-out among women between baseline and one-year follow-up was predicted by their EAS score on sociability, with higher scores associated with higher risk of dropping out. No other variables in the univariate analyses predicted drop-out. Sociability remained significant even after Bonferroni correction and after adjusting for all other variables. The polychoric correlation between baseline temperamental sociability and attrition at one-year follow-up was of a small to medium effect size (r =.20).

**Table 2:** Predictors of attrition from baseline to one- and 15- year follow-ups

	**One-year follow-up**				**15-year follow-up**			
	**Unadjusted**		**Adjusted**^‡^		**Unadjusted**		**Adjusted**^‡^	
**Predictor**	**OR**	**95% CI**	**OR**	**95% CI**	**OR**	**95% CI**	**OR**	**95% CI**
*Women:*								
Age ^†^	0.98	0.94-1.01	0.99	0.94-1.03	0.96*	0.93-0.99	0.99	0.96-1.02
Lives alone ^†^	1.52	0.86-2.70	1.44	0.77-2.68	1.93*	1.15-3.23	1.32	0.75-2.33
Family finances	0.91	0.76-1.08	0.95	0.76-1.17	0.87*	0.76-0.99	0.97	0.83-1.15
Education	0.87	0.73-1.03	0.84	0.68-1.04	0.59*	0.51-0.68	0.62*	0.52-0.74
Not working ^†^	1.35	0.94-1.96	1.32	0.86-2.03	2.01*	1.49-2.72	1.49*	1.06-2.10
Emotionality	1.05	0.88-1.25	1.06	0.84-1.33	1.03	0.90-1.18	0.98	0.82-1.16
Sociability	1.45*	1.20-1.75	1.56*	1.26-1.94	0.91	0.80-1.04	1.01	0.87-1.19
Activity	1.08	0.91-1.28	1.05	0.86-1.28	0.85*	0.75-0.97	0.95	0.82-1.11
Partner support	1.04	0.86-1.26	1.07	0.85-1.35	0.94	0.82-1.08	1.02	0.86-1.22
Emotional support from friends and family	0.97	0.81-1.15	0.86	0.70-1.05	0.88	0.77-1.01	0.93	0.79-1.08
Chronic stressors	1.00	0.84-1.19	1.03	0.81-1.31	1.00	0.87-1.14	1.01	0.84-1.21
HSCL	1.02	0.86-1.21	0.92	0.70-1.19	1.09	0.95-1.24	0.92	0.75-1.13
*Children:*								
Activity	1.10	0.92-1.31	0.98	0.80-1.20	1.02	0.90-1.17	0.98	0.84-1.15
Sociability	1.05	0.88-1.25	1.00	0.81-1.22	0.94	0.82-1.07	0.94	0.80-1.10
Emotionality	1.01	0.84-1.20	1.04	0.85-1.27	1.09	0.96-1.25	1.16	1.00-1.36
Shyness	0.96	0.81-1.14	0.95	0.77-1.17	1.05	0.92-1.20	0.94	0.80-1.10

Predictors are standardized scores from the baseline questionnaire. N = 913.

* Significant at p <.05 before Bonferroni correction, Bold font = significant at p < .05 after Bonferroni correction. ^‡^ N = 861 in the multiple regression analysis. (Partner support is left out of the multiple analysis reported here, because this reduced N to 779.) ^†^ unstandardized score.

Table 2 also shows that psychological distress, child’s temperament, chronic stressors, and women’s support from partner and friends at baseline did not predict attrition at 15-year follow-up. Several socio-demographic variables (low age, living alone, financial problems, low educational level, and not working) predicted attrition between baseline and 15-year follow-up. In addition, low temperamental activity level predicted long-term attrition. When adjusting for all variables, only low educational level and not working remained significant. Low educational level was the only significant predictor of long-term attrition after Bonferroni correction. The polychoric correlation between baseline educational level and attrition at 15-year follow-up was r = −.30, which is a medium effect size.

### Baseline associations between HSCL and other variables

The associations between mental health and other variables at baseline were not significantly different among those who stayed in the TOPP study and those who dropped out (see Table 3). The mean discrepancy between correlation coefficients for participants and non-participants were 0.05 and 0.04 at one-year and 15-year follow-up, respectively.

**Table 3:** Baseline correlations among participants and non-participants at one-year and 15- year follow-ups

**Predictors of HSCL**	**Drop-outs at one- year follow-up N = 155**	**Participants at one- year follow-up N = 758**	**Drop-outs at 15-year follow-up N = 514**	**Participants at 15- year follow-up N = 399**
*Women:*				
Age	-.12	-.17**	-.14**	-.18**
Alone with child	.19*	.12**	.16**	.09
Family finances	-.33**	-.28**	-.29**	-.28**
Education	-.15	-.18**	-.20**	-.13**
Not working	.16	.14**	.14**	.14**
Emotionality	.65**	.55**	.53**	.62**
Sociability	-.16*	-.19**	-.22**	-.13*
Activity	.00	-.01	-.01	-.01
Partner support	-.46**	-.37**	-.38**	-.40**
Emotional support from friends and family	-.28**	-.34**	-.33**	-.32**
Chronic stressors	.55**	.54**	.52**	.56**
*Children:*				
Activity	-.04	-.05	-.04	-.05
Sociability	-.04	.03	.05	-.02
Emotionality	.33**	.22**	.26**	.20**
Shyness	.11	.07*	.07	.09

* p < .05, ** p < .01. None of the correlations between HSCL and other variables were significantly different among those who later stayed in the study and those who dropped out.

### The simulation study

Estimated means and associations with 95% confidence intervals are reported as summary results from the 500 generated samples, as recommended by Maldonado and Greenland [44]. In addition, information about coverage is provided. This is the ratio of samples that had an estimated value with a 95% confidence interval containing the population value [21,22]. Regarding regression estimates, results are also provided for the ratio of samples that rejected the false null hypothesis of zero association between the two study variables, *Follow-up outcome* and *Baseline predictor*[21,22].

The results from the simulation study are shown in Tables 4, 5, and 6. As Table 4 shows, estimated means on *Follow-up outcome* became increasingly biased with higher attrition rates and also with stronger dependency between *Liability of dropping out* and *Follow-up outcome* (The effect of dependency between *Liability of dropping out* and *Baseline predictor* was minor). At a 50% attrition rate and a weak (β =.10) [43] dependency between *Liability of dropping out* and *Follow-up outcome,* the mean estimate was already borderline significantly different from the population mean (the 95% confidence interval was at a tangent to the population value). Furthermore, only 54% of the 500 randomly drawn samples yielded a 95% confidence interval that contained the true population mean under this condition. When the dependency between *Liability of dropping out* and *Follow-up outcome* reached a small to medium effect size, the mean estimate was significantly different from the population mean, and only 5.4% of the samples provided a 95% confidence interval containing the population mean, at a 50% attrition rate. At a medium dependency between *Liability of dropping out* and *Follow-up outcome,* none of the 500 samples provided 95% confidence intervals containing the true value, at a 50% attrition rate. These effects were weaker when the attrition rate was 30% and stronger when the attrition rate was 70%.

**Table 4:** Estimated means of follow-up variable; population mean = 0.00

		**30% attrition rate**		**50% attrition rate**		**70% attrition rate**	
**Dependency**		**Estimate (95% C.I.)**	**95% coverage**	**Estimate (95% C.I.)**	**95% coverage**	**Estimate (95% C.I.)**	**95% coverage**
**y**	**x**						
.00	.00	.00 (−.08,.08)	94	.00 (−.08,.08)	94	.00 (−.12,.12)	93
.10	.00	-.05 (−.13,.03)	75	-.08 (−.16,.00)	54	-.12 (−.24,.00)	47
.20	.00	-.10 (−.18,-.02)	22	-.16 (−.24,-.08)	5.4	-.23 (−.35,-.11)	3
.30	.00	-.15 (−.23,-.07)	2.6	-.24 (−.32,-.16)	0	-.35 (−.47,-.23)	0
.40	.00	-.20 (−.28,-.12)	0	-.32 (−.40,-.24)	0	-.46 (−.56,-.36)	0
.10	.10	-.06 (−.14,.02)	70	-.09 (−.17,-.01)	48	-.13 (−.25,-.01)	39
.20	.10	-.11 (−.19,-.03)	18	-.17 (−.25,-.09)	3.8	-.24 (−.36,-.12)	1
.30	.10	-.16 (−.24,-.08)	2	-.25 (−.33,-.17)	0	-.36 (−.48,-.24)	0
.40	.10	-.21 (−.29,-.13)	0	-.33 (−.41,-.25)	0	-.48 (−.58,-.38)	0
.20	.20	-.11 (−.19,-.03)	14	-.18 (−.36,-.10)	2.6	-.25 (−.37,-.13)	1
.30	.20	-.16 (−.24,-.08)	1	-.26 (−.34,-.18)	0	-.37 (−.49,-.25)	0
.40	.20	-.21 (−.29,-.13)	0	-.33 (−.41,-.25)	0	-.49 (−.59,-.39)	0
.30	.30	-.17 (−.25,-.09)	1	-.26 (−.34,-.18)	0	-.38 (−.60,-.16)	0
.40	.30	-.22 (−.30,-.14)	0	-.34 (−.42,-.26)	0	-.50 (−.60,-.40)	0
.40	.40	-.22 (−.30,-.14)	0	-.35 (−.43,-.28)	0	-.51 (−.61,-.41)	0

Dependency = direct effect (β) of study variables on *Liability of attrition*. Y is dependency between attrition and *Follow-up outcome* (path c in Figure 1), x is dependency between attrition and *Baseline predictor* (path b in Figure 1). N in the original sample is 1000. The association between *Baseline predictor* and *Follow-up outcome* is β = .10. 95% C.I. = 95% confidence interval. Parameter estimates and 95% C.I.s are average results over the 500 generated samples. 95% coverage = proportion of the 500 samples where the 95% confidence intervals contain the population value. The dependency between study variables and the continuous *Liability of dropping out* is reported in βs. The corresponding ORs of the associations between study variables and a dichotomized drop-out variable would be about the following (depending on attrition rate): β =.00 equals OR =1; β =.10 equals OR = 1.22; β =.20 equals OR =1.49; β =.30 equals OR =1.82; β =.40 equals OR = 2.23.

**Table 5:** Estimated associations between baseline predictor and follow-up outcome; population β = .10

		**30% attrition rate**		**50% attrition rate**		**70% attrition rate**	
**Dependency**		**Estimated β (95% C.I.)**	**95% coverage (% Sig)**	**Estimated β (95% C.I.)**	**95% coverage (% Sig)**	**Estimated β (95% C.I.)**	**95% coverage (% Sig)**
**y**	**x**						
.00	.00	.10 (.02,.18)	96% (76)	.10 (.02,.18)	93% (61)	.10 (−.02,.22)	94% (44)
.10	.00	.10 (.02,.18)	95% (77)	.10 (.02,.18)	93% (62)	.10 (−.02,.22)	93% (47)
.20	.00	.10 (.02,.18)	95% (74)	.10 (.02,.18)	93% (60)	.10 (−.02,.22)	93% (43)
.30	.00	.10 (.02,.18)	94% (75)	.10 (.02,.18)	94% (61)	.10 (−.02,.22)	94% (41)
.40	.00	.10 (.02,.18)	94% (73)	.10 (.02,.18)	95% (58)	.09 (−.03,.21)	95% (37)
.10	.10	.09 (.01,.17)	95% (71)	.09 (.01,.17)	93% (57)	.09 (−.03,.21)	94% (38)
.20	.10	.09 (.01,.17)	94% (66)	.09 (.01,.17)	92% (51)	.08 (−.04,.20)	92% (31)
.30	.10	.08 (.00,.16)	91% (59)	.08 (.00,.16)	90% (39)	.07 (−.05,.19)	91% (27)
.40	.10	.08 (.00,.16)	86% (51)	.07 (−.01,.15)	86% (34)	.06 (−.06,.18)	88% (24)
.20	.20	.08 (.00,.16)	90% (56)	.07 (−.01,.15)	88% (38)	.07 (−.05,.19)	89% (24)
.30	.20	.07 (−.01,.15)	82% (41)	.06 (−.02,.14)	81% (23)	.05 (−.07,.17)	83% (16)
.40	.20	.05 (−.03,.13)	70% (27)	.04 (−.04,.12)	66% (15)	.03 (−.09,.15)	68% (11)
.30	.30	.05 (−.03,.13)	66% (23)	.03 (−.07,.13)	61% (12)	.02 (−.10,.14)	65% (10)
.40	.30	.03 (−.05,.11)	41% (11)	.01 (−.09,.11)	37% (8)	-.01 (−.13,.11)	43% (7)
.40	.40	.00 (−.08,.08)	15% (7)	-.03 (−.13,.07)	13% (13)	-.05 (−.19,.07)	20% (15)

Population β is the effect of the baseline predictor on the later outcome in the population. Dependency = direct effect (β) of study variables on *Liability of attrition*. Y is dependency between attrition and *Follow-up outcome* (path c in Figure 1), x is dependency between attrition and *Baseline predictor* (path b in Figure 1). N in the original sample is 1000. 95% C.I. = 95% confidence interval. Parameter estimates and 95% C.I.s are average results over the 500 generated samples. 95% coverage = proportion of the 500 samples where the 95% confidence intervals contain the population value. % Sig = proportion of the 500 samples where the false null hypothesis of zero association between *Baseline predictor* and *Follow-up outcome* is rejected at the .05 level. The dependency between study variables and the continuous *Liability of dropping out* is reported in βs. The corresponding ORs of the associations between study variables and a dichotomized drop-out variable would be about the following (depending on attrition rate): β =.00 equals OR =1; β =.10 ≈ OR = 1.22; β =.20 ≈ OR =1.49; β =.30 ≈ OR =1.82; β =.40 ≈ OR = 2.23.

**Table 6:** Estimated associations between baseline predictor and follow-up outcome; population β = .30

		**30% attrition rate**		**50% attrition rate**		**70% attrition rate**	
**Dependency**		**Estimated β (95% C.I.)**	**95% coverage (% Sig)**	**Estimated β (95% C.I.)**	**95% coverage (% Sig)**	**Estimated β (95% C.I.)**	**95% coverage (% Sig)**
**y**	**x**						
.00	.00	.30 (.24,.36)	95% (100)	.30 (.22,.38)	94% (100)	.30 (.20,.40)	95% (100)
.10	.00	.30 (.24,.36)	95% (100)	.30 (.22,.38)	94% (100)	.30 (.20,.40)	94% (100)
.20	.00	.30 (.24,.36)	94% (100)	.30 (.22,.38)	94% (100)	.30 (.20,.40)	94% (100)
.30	.00	.29 (.23,.35)	93% (100)	.29 (.21,.37)	93% (100)	.29 (.19,.39)	93% (100)
.40	.00	.29 (.23,.35)	88% (100)	.29 (.21,.37)	89% (100)	.28 (.18,.38)	89% (100)
.10	.10	.29 (.23,.35)	95% (100)	.29 (.21,.37)	94% (100)	.29 (.19,.39)	94% (100)
.20	.10	.29 (.21,.37)	91% (100)	.28 (.20,.36)	91% (100)	.28 (.18,.38)	90% (100)
.30	.10	.28 (.20,.36)	83% (100)	.27 (.19,.35)	82% (100)	.27 (.17,.37)	84% (100)
.40	.10	.25 (.18,.33)	73% (100)	.26 (.18,.34)	69% (100)	.25 (.15,.35)	70% (100)
.20	.20	.28 (.20,.36)	83% (100)	.29 (.21,.37)	80% (100)	.26 (.16,.36)	85% (100)
.30	.20	.26 (.18,.34)	69% (100)	.25 (.17,.33)	65% (100)	.24 (.14,.34)	68% (99)
.40	.20	.25 (.18,.33)	44% (100)	.23 (.15,.31)	38% (100)	.22 (.10,.34)	42% (97)
.30	.30	.24 (.16,.32)	43% (100)	.23 (.15,.31)	33% (100)	.21 (.09,.33)	41% (95)
.40	.30	.22 (.14,.30)	16% (100)	.20 (.12,.28)	10% (99)	.18 (.06,.30)	15% (86)
.40	.40	.19 (.11,.27)	3% (100)	.15 (.07,.23)	1% (92)	.13 (.01,.25)	2% (59)

Population β is the effect of the baseline predictor on the later outcome in the population. Dependency = direct effect (β) of study variables on *Liability of attrition*. Y is dependency between attrition and *Follow-up outcome* (path c in Figure 1), x is dependency between attrition and *Baseline predictor* (path b in Figure 1). N in the original sample is 1000. 95% C.I. = 95% confidence interval. Parameter estimates and 95% C.I.s are average results over the 500 generated samples. 95% coverage = proportion of the 500 samples where the 95% confidence intervals contain the population value. % Sig = proportion of the 500 samples where the false null hypothesis of zero association between *Baseline predictor* and *Follow-up outcome* is rejected at the .05 level. The dependency between study variables and the continuous *Liability of dropping out* is reported in βs. The corresponding ORs of the associations between study variables and a dichotomized drop-out variable would be about the following (depending on attrition rate): β =.00 equals OR =1; β =.10 ≈ OR = 1.22; β =.20 ≈ OR =1.49; β =.30 ≈ OR =1.82; β =.40 ≈ OR = 2.23.

Tables 5 and 6 show that regression estimates, as opposed to mean estimates, were only weakly affected by attrition rate. In addition, regression estimates were only minimally affected by the dependency between *Liability of dropping out* and *Follow-up outcome* Even moderate to strong dependency between *Liability of dropping out* and *Follow-up outcome* had only very weak effects on regression estimates. In addition, the proportion of samples that provided a regression estimate with a 95% confidence interval containing the true population value (95% coverage) was only weakly affected by increasing dependency between *Liability of dropping out* and *Follow-up outcome* However, when *Liability of dropping out* was dependent on *Baseline predictor* in addition to *Follow-up outcome,* regression estimates and their 95% coverages were affected. This was true at 30%, 50%, and 70% attrition rates.

## Discussion

Findings from the TOPP study showed that those who stayed compared to those who dropped out over the15-year period differed in baseline educational level but not in regard to baseline mental health and relationship variables. Furthermore, the two groups did not differ significantly regarding associations between variables. The results from the simulation study showed that mean estimates became substantially biased even at relatively weak dependencies between follow-up variables and attrition, whereas estimates of associations between variables were more robust to dependencies between attrition and study variables. In addition, mean estimates, but not regression estimates, were strongly affected by attrition rate. The results are more thoroughly discussed below.

Temperamental sociability was a significant predictor of short-term attrition (baseline to one-year follow-up), in that high scores on sociability predicted higher chances of dropping out. Apart from a study showing that antisocial personality predicted having died at follow-up [5], there are few studies on adult personality and attrition from population-based studies. Our finding shows that psychological variables other than psychopathology can be important for understanding attrition.

In a long-term perspective (baseline to 15-year follow-up), educational level predicted drop-out. The sample became moderately biased towards having more well-educated participants over time, which is in accordance with previous attrition studies finding that socio-demographic variables predict drop-out [2,4,9-11].

An important question when examining long-term attrition was whether those who stayed and those who dropped out differed on psychological and social variables at baseline. Some population-based studies have found weak to moderate dependencies between adult psychiatric diagnosis and attrition after adjusting for other variables [9,10]. In studies where self-rating measures were used, psychological distress was found to have no effect or a weak to moderate effect after adjusting for other variables [2,4]. The results of the present study are more in accordance with the latter, as psychological characteristics of neither the women nor children nor qualities of the spouse/partner relationship predicted long-term attrition. Slightly divergent results may be due to different measures of psychological distress. Our results are also in accordance with previous research showing no associations between baseline child characteristics, such as temperament and anxiety, and attrition in population-based studies [18,19].

Even though baseline sociability and educational level predicted attrition, the baseline associations between these variables and mental health were the same among those who later dropped out and those who remained in the study. Of the 15 correlations between psychological distress and other variables examined at baseline, none were significantly different for participants and non-participants at one- or 15-year follow-up. The current findings thus show that even if those who stay and those who drop out of a study differ regarding mean levels of some variables, estimates of associations can be robust to such differences.

The current simulation study provided information about effects of attrition dependent on follow-up as well as baseline variables. The results showed that mean estimates became increasingly biased as attrition rates increased. At 50% and 70% attrition rates, mean estimates became extremely biased, even at weak dependencies between attrition and follow-up variables. Mean estimates became increasingly biased as the dependency between risk of attrition and the study variable got stronger. Therefore, mean estimates from longitudinal studies should be interpreted with caution, even when attrition is only weakly dependent on the variables of interest. These results are in accordance with findings from a study of the effect of selective enrolment in a large population-based study of pregnant women [8]. Nilsen and colleagues used information about medical conditions among non-responders from a national register and concluded that mean estimates of age, number of cigarettes smoked, birth weight, and other medical variables were biased among participants because of selective participation in the study [8].

The simulation study further showed that regression estimates were only minimally affected by attrition rate. Regression estimates and their 95% coverage were very similar at both lower and higher attrition rates. In addition, the degree of dependency between attrition and the follow-up variable had only weak effects on regression estimates and their 95% coverages. This was the case both when the population association between predictor and outcome was weak and when it was moderate. Naturally, the proportion of samples that rejected the false null hypothesis of a zero association between the two study variables was higher with stronger population associations. This proportion did not decrease notably as the dependency between attrition and follow-up variables increased. The effect of attrition on estimates of associations between variables thus seemed to be limited to the effect of reduced N when attrition was only dependent on follow-up variables.

However, when attrition became increasingly dependent on both baseline and follow-up variables, the regression estimates were seriously biased, and the 95% coverage dropped dramatically. For weak population associations between variables, the proportion of samples that succeeded in rejecting the false null hypothesis also decreased when attrition became increasingly dependent on both baseline and follow-up variables.

The current results indicate that attrition related to both baseline and follow-up variables has far worse consequences for regression estimates than attrition that is only related to follow-up variables. Being able to account for attrition related to baseline variables can thus reduce the negative consequences of selective attrition on regression estimates. Modern techniques for handling missing data (e.g. full information maximum likelihood and multiple imputation methods) are effective in adjusting for missingness that is dependent on variables with information from all participants [20]. In longitudinal studies with attrition, the researcher typically has information on baseline variables from all participants, but lack of information on follow-up variables from those who have dropped out. The current results suggest that using such techniques to account for attrition related to baseline variables can reduce the negative effects of selective attrition on regression estimates even if these techniques do not account for attrition related to follow-up variables.

Graham and Donaldson [24] reported from their simulation study that non-random attrition affected estimation of the effect of an intervention. They concluded that correlation estimates were biased when attrition differed between the control group and the intervention group, but that correlation estimates based on complete cases were unbiased when attrition was the same in both groups, even though attrition was dependent on measured and unmeasured variables. They did not compare different degrees of dependency between attrition and the study variables. Our results thus extend their findings by showing effects of attrition with different degrees of dependency on baseline and follow-up variables.

### Limitations

Although the real life study had several strengths-being population-based, extending over a long period of time, and having a relatively large number of participants-there are also some limitations.

First, individuals with the highest levels of mental health problems and alcohol use tend to participate less often than others in population-based studies [12]. Even though the current results indicate that samples in long-term longitudinal studies may be comparable to those in cross-sectional studies, both kinds of studies face challenges regarding generalizability to persons with high levels of mental health problems. Second, staff at the health care centers organized the data collection at the first three time points, whereas the questionnaires were distributed by mail at later waves. Differences in data collection methods may have influenced attrition in the short-term compared to long-term perspective. Third, some of the measures showed somewhat low reliability, and this may have affected the results. Fourth, the results of this attrition study may be generalizable only to questionnaire studies. Thus, other kinds of studies, such as those employing interviews, need to be examined separately. Fifth, some argue that the Bonferroni correction produces too conservative p-thresholds and therefore too high risk of type II errors [45]. Working status was a significant predictor of long-term attrition before but not after Bonferroni correction. However, there were no other differences before and after Bonferroni correction in the adjusted solutions. Bonferroni correcting of the results thus had only minimal impact on our conclusions. Moreover, the power analysis conducted showed that relatively small effects in the population could be detected with high probabilities. Thus, the non-findings in the real life study are probably not a result of low statistical power. Sixth, attrition from the TOPP study was mainly due to refusal to participate. Other reasons for attrition from population-based studies, such as death or failure to locate, may have other consequences for generalizability of findings. Finally, the sample size used in the current simulation study was similar to the baseline sample size in the real life study. Different sample sizes can provide different confidence intervals and thus different results regarding statistical significance. Therefore, further simulation studies are needed to examine the effect of attrition under several different conditions.

## Conclusions

Together, the findings from the TOPP study and the simulation study suggest that even if estimates of means can be seriously biased in longitudinal studies, estimates of associations seem to be far more robust to selective attrition. Attrition rate affected mean estimates but not regression estimates.

Even at moderate to strong dependencies between attrition and follow-up variables, estimates of associations between variables seem to be generalizable. However, when attrition is dependent on both baseline and follow-up variables, regression estimates tend to be biased. Researchers should therefore use modern missing data techniques to account for attrition related to baseline variables to reduce the negative effects of attrition on regression estimates.

These are important findings both because attrition is common in longitudinal studies and because public health research often aims to study associations between risk/protective factors and health.

## Competing interests

The authors declare that they have no competing interests.

## Authors’ contributions

KG performed the statistical analyses. KG drafted the manuscript. TvS, ER, and EK contributed to designing the questionnaires and collecting the data. All authors contributed to the design of this specific study, the interpretation of results, and helped to draft or critically revise the manuscript. All authors read and approved the final manuscript.

## Pre-publication history

The pre-publication history for this paper can be accessed here:

http://www.biomedcentral.com/1471-2458/12/918/prepub
